# A Multi-Omics Framework for Decoding Disease Mechanisms: Insights From Methylmalonic Aciduria

**DOI:** 10.1016/j.mcpro.2025.100998

**Published:** 2025-05-26

**Authors:** Jianbo Fu, Vito R.T. Zanotelli, Cedric Howald, Nylsa Chammartin, Ilya Kolpakov, Ioannis Xenarios, D. Sean Froese, Bernd Wollscheid, Patrick G.A. Pedrioli, Sandra Goetze

**Affiliations:** 1 Department of Health Sciences and Technology, Institute of Translational Medicine, Swiss Federal Institute of Technology, ETH Zurich, Zurich, Switzerland; 2 Swiss Institute of Bioinformatics (SIB), Lausanne, Switzerland; 3 ETH PHRT Swiss Multi-Omics Center (SMOC), Zurich, Switzerland; 4 Division of Metabolism and Children’s Research Center, University Children’s Hospital Zurich, University of Zurich, Zurich, Switzerland; 5 EPFL PHRT Swiss Multi-Omics Center (SMOC), Lausanne, Switzerland; 6 Health 2030 Genome Center, Foundation Campus Biotech, Geneva, Switzerland

**Keywords:** methylmalonic aciduria, multi-omics data integration, quantitative trait loci, pQTL, correlation network analysis, module analysis, gene set enrichment analysis, transcription factor enrichment analysis

## Abstract

The diverse perspectives offered by multi-omics data analysis can aid in identifying the most relevant molecular pathways involved in disease processes, and findings in one layer can substantiate findings in other layers of information. Integrating data from multiple omics sources is becoming increasingly important to improve disease diagnosis and treatment, especially for conditions with complex and poorly understood underlying pathomechanisms. Methylmalonic aciduria (MMA), an inherited metabolic disorder, serves as an illustrative example of such a disease with poorly understood pathogenesis for which published multi-omics data are readily available. Reusing these FAIR data, obtained from the multi-omics digitization of 230 individuals (210 patients with MMA and 20 controls), we pursued advanced data integration and analysis strategies to integrate different levels of biological information, combining genomic, transcriptomic, proteomic, and metabolomic profiling with biochemical and clinical data, with the aim of elucidating molecular perturbations in individuals affected by MMA. The analysis of protein-quantitative trait loci highlighted the importance of glutathione metabolism in the pathogenesis of MMA. This finding was supported by correlation network analyses that integrated proteomics and metabolomics data, alongside gene set enrichment and transcription factor analyses based on disease severity from transcriptomic data. The correlation network analysis also revealed that lysosomal function is compromised in patients with MMA, which is critical for maintaining metabolic balance. Our research introduces a comprehensive data analysis framework that effectively addresses the challenge of prioritizing disruptions in molecular pathways by accumulating evidence from multiple omics levels.

## Highlights

• Integrative multi-omics helps identify disease mechanisms in complex disorders.
• pQTL analysis in MMA reveals impact of genetic variation on disease-related proteins.
• Integrative analysis identifies glutathione metabolism as key in MMA pathogenesis.
• The analysis pipeline is shared as a Jupyter notebook for easy reuse.

## Graphical Abstract

**Figure:**
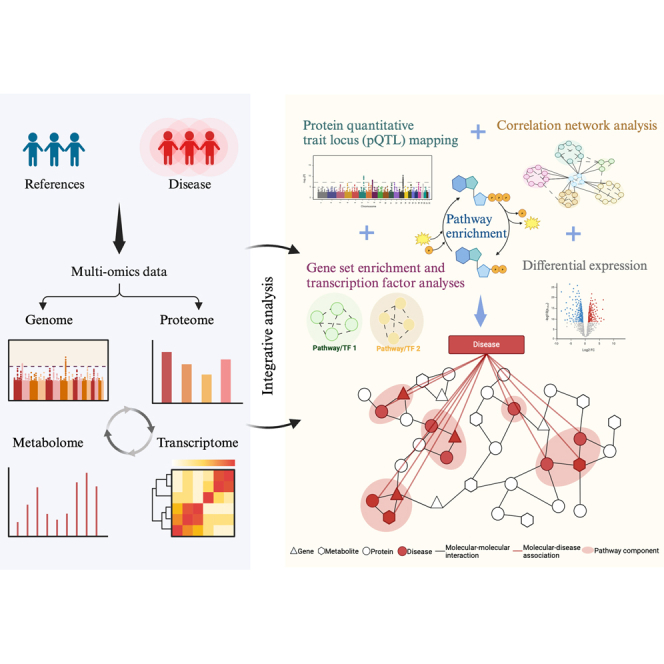

## In Brief

Integrative multi-omics has the potential to improve disease diagnosis and treatment by uncovering underlying molecular pathways. Using data from individuals with methylmalonic aciduria (MMA), genomic, transcriptomic, proteomic, and metabolomic profiling was integrated with clinical data to elucidate the pathogenesis of MMA. This revealed the relevance of glutathione metabolism in MMA, a finding that was corroborated by evidence from multi-molecular layers. The analytical framework we provide has broad applicability to other diseases with complex pathomechanisms.

Biological processes are inherently complex and orchestrated by billions of molecules. The interaction and crosstalk between these biomolecules enable the regulation of essential processes, including cell division and growth, gene expression, signal transduction, and metabolism. In disease, these processes can become dysregulated, leading to pathological conditions. Therefore, a better understanding of the nature of the connectivity and interactions of biomolecules is crucial for identifying underlying disease mechanisms and the development of targeted therapies.

In recent years, increases in speed and reductions in costs of multi-omic technologies have made it possible for researchers to generate multi-omics data on a larger scale and gain a more comprehensive understanding of complex biological networks (1, 2, 3, 4, 5, 6). Within this framework, whole exome sequencing (WES) and whole genome sequencing (WGS) provide information about genetic variations and are now standard diagnostic tools for a wide range of diseases, including cancer and rare genetic disorders (7, 8, 9, 10). This is transforming healthcare by enabling faster, more precise diagnostics and personalized treatment plans (11). In contrast to the genome, which is stable, the expression of mRNAs and proteins, as well as their associated metabolites, varies in cells, tissues, and organs and also over time. The analysis of the mRNA transcriptome by RNA sequencing provides comprehensive insights into gene expression patterns, revealing which genes are active or repressed under specific conditions, as well as information about alternative splicing events. Protein levels can fluctuate in response to external stimuli, eventually leading to different biochemical interactions and cellular responses. The analysis of the proteome, therefore, provides a link between the genotype and phenotype. Metabolites are intermediates of biochemical reactions and play a crucial role in metabolic pathways and signaling events. The metabolome, which comprises the collection of all metabolites, provides a direct reflection of an organism's biochemical activities and metabolic state. Unlike genomics, transcriptomics, or proteomics, metabolomics captures a broad range of dynamic metabolic states, offering a dynamic view of the phenotype.

Interpreting the various layers of omics data is challenging, particularly when the functions of many identified biomolecules are unknown. Systematic co-expression analysis helps address this complexity by clustering biomolecules into modules based on global expression levels and correlation estimates (12, 13, 14). These modules enable researchers to understand biomolecular interactions and predict potential functions, as biomolecules with similar roles often exhibit correlated expression patterns. In recent years, various co-expression analysis software packages have been developed, including Weighted Gene Co-expression Network Analysis (WGCNA) (15), Co-expression Modules Identification Tool (CEMiTool) (16, 17), and RNA-seq-based tools like coseq (18). The integration of multi-omics data over the different layers harbors further potential to elucidate functional linkage, unravel underlying disease mechanisms, identify robust biomarkers, and formulate individualized treatment modalities. Integrating quantitative trait loci (QTLs) across multidimensional omics data represents an emerging approach for examining the impacts of genetic variants across diverse omics modalities (19, 20, 21). QTL analysis serves as a valuable tool in bridging the connection between genes and the phenotypic traits that result from them. It is especially useful for elucidating the genetic basis of complex traits that are influenced by multiple genes or environmental factors (19, 22). Protein quantitative trait locus (pQTL) analysis combines genome-wide genotyping data with quantitative proteomics measurements to map genetic loci that influence protein abundance levels. pQTL studies can reveal both cis-acting variants (located within 1 MB of the encoding gene) and trans-acting variants (located elsewhere in the genome) that affect protein levels.

Here, we applied for the first time pQTL in combination with co-expression analysis on a multi-omics data set generated in the context of isolated methylmalonic aciduria (MMA) (2). MMA is a rare metabolic disorder characterized by the accumulation of methylmalonic acid in the body due to a disruption in the normal propionyl-CoA catabolic pathway. Biochemically, this condition results from the inability to convert methylmalonyl-CoA to succinyl-CoA, a critical step in the breakdown of branched-chain amino acids, odd-chain fatty acids, and cholesterol. Defects in *MMUT*, *MMAA*, and *MMAB* are the most frequent genetic causes of MMA, affecting the mitochondrial enzyme methylmalonyl-CoA mutase (MMUT) or its cofactor adenosylcobalamin (AdoCbl), derived from vitamin B12 (cobalamin, Cbl) (23, 24). The function of MMUT has been the subject of extensive study; however, the principal metabolic disturbances and underlying mechanisms of action in MMA remain unresolved and a significant fraction of affected individuals remain undiagnosed (23, 25). One of the main reasons for this, next to poor pathomechanistic understanding, is the often-unclear genotypephenotype relationship (25).

Here, we first conducted a protein quantitative trait locus (pQTL) analysis, which revealed loci enriched for functional traits. This was followed by enrichment analyses to identify proteins with genome-wide significant pQTLs, and correlation network analyses on proteomics and metabolomics data to identify modular proteins/metabolites significantly associated with MMA. To support our findings, we performed a gene set enrichment analysis (GSEA) (26). and a transcription factor (TF) enrichment analysis (27) on transcriptomic data. Our integrative approach revealed pathways such as glutathione metabolism, lysosomal function, and TCA cycle regulation to be of relevance in MMA.

## Experimental Procedures

### Experimental Design and Statistical Rationale

The presented analysis approach is demonstrated based on an MMA dataset described previously (2). To briefly recapitulate, the dataset is based on biobanked samples (total n = 210) of a methylmalonic aciduria cohort. Phenotypic data of the patients used for analyses are provided in supplemental Table S1. The patient fibroblast samples discussed in this manuscript include n = 120 MMA samples with a complete loss of MMUT enzyme activity (mut^0^), n = 30 samples with a partial reduction of MMUT enzyme activity (mut^-^), and n = 60 with normal MMUT enzymatic activity. The cohort was collected over a period of more than 25 years accounting for the fact that the occurrence of MMA is rare (supplemental Fig. S1). The rare occurrence of MMA is also the reason why sample numbers are not balanced for disease severity. However, the cohort is balanced in gender and sample selection over time (supplemental Fig. S1). Our analysis additionally includes 20 fibroblast samples collected from unaffected individuals. Due to the limited availability of healthy/unaffected donors, the unaffected reference samples do not match the amount of MMA patient samples. Due to the limited amount of biological material that had to be split between the different omics technologies, no replicate analysis was performed for the patient samples. Regarding quality control for sampling and MS performance over time, we measured two pools of patient samples as technical replicates (n = 10 for each of the two pools). Retention time peptides (iRTs) (Biognosys) were used to check for retention time shifts. We randomized the sample processing for proteomics in blocks of eight, taking into consideration a balance between disease types and control samples. All other factors within a block were randomized. This was done to keep the variability between the different sample processing batches low. A spectral library was generated and used for analysis (2).

### Cohort and Patient-Derived Fibroblast Samples

Primary fibroblast samples from MMA patients and associated disease-related information, including clinical and diagnostic data, were collected between 1989 and 2015. The collection and use of these fibroblasts were conducted under the ethical approval granted by the Ethics Committee of the Canton of Zurich (KEK-2014-0211, amendment: PB_2020-00053) and comply with the Declaration of Helsinki. After collection, primary fibroblasts were cultured using Dulbecco’s modified Eagle’s medium (DMEM; Gibco, Life Technologies) with 10% fetal bovine serum (Gibco) and antibiotics (GE Healthcare) (2). A frozen aliquot of each primary fibroblast culture was used for WGS, RNA-seq, and data-independent acquisition mass spectrometry (DIA-MS). Control samples were obtained from healthy individuals or donors with a biochemical defect whose diagnosis excluded MMA.

### Whole Genome Sequencing (WGS)

Genomic DNA was extracted using the QIAmp DNA Mini Kit (QIAGEN) according to the manufacturer’s instructions. Whole genome sequencing (WGS) libraries were prepared with the TruSeq DNA PCR-Free Library Kit (Illumina) using 1 μg of genomic DNA, following the provided protocol. The resulting genomic DNA libraries were quantified with the KAPA Library Quantification Complete Kit (Roche) as per the manufacturer's guidelines. Sequencing was performed on the NovaSeq 6000 platform (Illumina) in a 150-nucleotide paired-end configuration. After performing routine quality assessments of the sequencing data, small variants were identified using GATK version 4.0.9.0, following the recommended best practices (28).

### RNA-Sequencing

Total RNA was extracted using the Rneasy Plus Mini Kit (QIAGEN). RNA-seq libraries were prepared with the TruSeq Stranded mRNA-seq Kit (Illumina) using 200 ng of total RNA, following the manufacturer's instructions. Sequencing was conducted on the Illumina HiSeq 4000 platform with a 75-nucleotide paired-end configuration (supplemental Table S2).

### Sample Preparation for Protein Measurements by Mass Spectrometry

Samples were processed in blocks of eight, ensuring a balance between disease types and controls, with all other variables within each block randomized. Primary fibroblasts (∼1e6 cells per vial) were washed twice with ice-cold PBS (Gibco), resuspended in lysis buffer (Preomics) at a 1:1 ratio (vol pellet/vol lysis buffer), and incubated at 95 °C for 10 min. Samples were then sonicated using a vial tweeter (Hielscher Ultrasound Technology) at 4 °C for three cycles, with 100% amplitude and 80% power for 30 s. Subsequently, 100 μg of protein lysate was processed using the iST kit (Preomics), digested for 3 hours, and the purified peptides were resuspended in LC-Load buffer at a concentration of 1 μg/μl containing iRT peptides (Biognosys).

### DIA-MS Setup for Protein Measurements

For DIA-MS, samples were analyzed on a Q-Exactive HF mass spectrometer (Thermo Fisher Scientific). Mobile phase A was HPLC-grade water with 0.1% formic acid, while mobile phase B was HPLC-grade acetonitrile (ACN) containing 20% HPLC-grade water and 0.1% formic acid. Peptide separation was performed on a C18 EASY-Spray column (ES806, Thermo Fisher Scientific) with dimensions 150 μm × 150 mm, 2 μm, 100 Å, at 50°C. For LC-MS/MS, 2 μg of the sample was injected onto the column using an Easy-nLC 1200 HPLC system (Thermo Fisher Scientific) at a flow rate of 4 μl/min with 100% mobile phase A for 5 min. Peptides were eluted using a gradient of 2% to 8% mobile phase B over 4 min, 8% to 32% mobile phase B over 49 min, 32% to 60% mobile phase B in 1 min, and ramping to 98% mobile phase B in 1 min, at a flow rate of 2 μl/min.

For DIA acquisition on a Q-Exactive HF mass spectrometer, we utilized the high-resolution MS1-based quantitative data-independent acquisition (HRMS1-DIA) approach (3, 29). MS1 scans were conducted across an m/z range of 400 to 1210 at a resolution of 120,000, with an AGC target of 3e6 and a maximum injection time of 50 ms. MS/MS scans were performed at a resolution of 30′000, with an AGC target of 1e6 and an “Auto” maximum injection time. Precursor ions were isolated within a 15-Th window and fragmented using HCD with a normalized collision energy of 28. 54 MS/MS scan windows were defined, with an MS1 scan interspersed every 18 scans.

### DIA-MS Data Analysis

For DIA analysis, an in-house generated spectral library was used, the details of library generation are described elsewhere (2). In short, raw files were searched in Proteome Discoverer v2.4 (Thermo Fisher Scientific) against a Uniprot human library (release April 2018) together with standards and common contaminants. Precursor mass tolerance was set to 10 ppm, and fragment mass tolerance to 0.02 DA. For the identification in Spectronaut, a Qvalue (FDR) cutoff of 0.01 was applied to the precursor as well as to the protein level. Single hits were defined based on their stripped sequence. The average mass tolerance reported at the run level in Spectronaut was around 8 ppm for MS1 and at the fragment level below 20 ppm. Protease cleavage was defined as Trypsin (full) with a maximum of two miscleavages. The minimum peptide length was set to six amino acids. Carbamidomethylation (C) was set as static, oxidation (M), deamidation (N), phosphorylation (S,T,Y), and N-terminal acetylation as dynamic modifications. PSM site probability threshold was chosen to be 75. The spectral library raw files and the library tsv files can be accessed *via* MassIVE (MSV000088791). DIA data analysis was performed in Spectronaut v.12 (Biognosys) using standard parameters. The spectral library generated in Spectronaut from the Proteome Discoverer search results consisted of 74′378 peptides and 8′431 protein groups. Decoys were generated using mutated decoy generation methods. Duplicated spectra were excluded based on global performance. The MS1 area was selected for quantification, interference correction was performed. Quantification parameters were set to mean peptide quantity for major group quantity; the top three peptides were selected for protein quantity calculation. Data filtering was set to Q value sparse. Cross-run normalization was set to local. The Spectronaut normalized protein report was filtered to remove proteins with abundance values smaller than 1000 in more than 20% of the samples. The list of protein group (PG) abundances used for analysis is provided as supplemental Table S3.

### Measurements of Polar Metabolites

Metabolite profiling was performed as described elsewhere (2). In short, untargeted metabolite profiling was conducted using flow injection analysis on an Agilent 6550 QTOF instrument with negative ionization and high-resolution MS1 scanning. Samples were processed in seven randomized batches, and data was centroided and analyzed using Matlab. Missing values were filled by recursive extraction from the raw data, and ions were annotated by accurate mass and isotopic patterns using the HMDB v4.0 database (supplemental Table S4). For annotation, mass accuracy was set to ±0.001 Da, corresponding to a mass error of approximately 1 to 3 ppm depending on the exact m/z value. Observed isotopic peaks were required to match theoretical distributions within a relative abundance tolerance of ±20%. Under negative ionization conditions, we focused primarily on deprotonated [M–H]^-^ ions and generated a list of putative metabolites from HMDB v4.0, that included known adducts and potentially fluorinated forms introduced by NH_4_F. Multiple charge states or ion pairs for the same metabolite were not combined, but redundant signals (adducts, isotopomers, and unmatched peaks) were removed to ensure that each annotated ion corresponded as closely as possible to a single molecular entity.

### pQTL Analysis

The cis-pQTL analysis was restricted to gene coding regions and lncRNAs to prioritize genetic variants with clear functional and regulatory relevance. The analysis was performed using version 1.1 of QTLtools, with technical parameters set as recommended in (30). Specifically, the cis-window was set to 1 MB, and the number of principal components of the genotype matrix and the quantification matrix to use as covariates were chosen to maximize the number of discoveries. Adjusted *p*-values were calculated using 1000 permutations, and a stringent cutoff of 5e-8 was applied to identify statistically significant cis-pQTLs for follow-up analysis.

### Correlation Network Analysis

Co-expression module analysis was executed using the CEMiTool package (16). To identify highly connected proteins (hub proteins) within each module, protein-protein interactions (PPIs) were integrated with coexpression data. Subsequently, proteins from these coexpressed modules were searched within the STRING 11.0 PPI database to identify all possible interactions. The protein network was created using the ‘plot_interaction’ function in CEMiTool, highlighting proteins with high degrees of connectivity as hub proteins in both the coexpression module and the PPI network. In metabolomic data, the degree of connectivity was directly used to determine highly connected metabolites (hub metabolites) for each coexpression module. To identify "central-hub" proteins, we used degree centrality (number of network connections) and selected proteins in the top percentile of their module’s connectivity distribution. This data-driven cutoff, tailored to the network’s intrinsic structure, ensured only highly connected proteins were classified as central hubs, avoiding arbitrary thresholds.

To identify metabolic pathways enriched within each module, we utilized Metascape software for proteins (31), performing enrichment analysis with the default parameters at each stage. Metascape conducted hierarchical clustering of enriched terms, displaying the most significant term within each cluster as the representative term in the enrichment results. For metabolites, pathway enrichment analysis of identified metabolite lists was carried out using the Hypergeometric test function in MetaboAnalyst V6.0 (https://www.metaboanalyst.ca/) (32). The KEGG library was selected as the reference pathway library for this analysis. For all pathway enrichment calculations, the sets of identified proteins/metabolites were used as a background.

### Disease Severity Correlation-Based GSEA

For Gene Set Enrichment Analysis (GSEA), we used the clinical severity score (CSS) capturing disease severity (supplemental Table S1) as calculated in Forny *et al*. (2). Using the transcriptomic data, we applied Kendall correlation to associate genes with CSS scores. Based on the resulting correlation coefficients with the CSS, we performed GSEA to identify significantly enriched pathways related to disease progression.

### Transcription Factor Analysis

For transcription factor (TF) enrichment analysis, we utilized the enrichR package (33) to access the ChEA3 database (27), allowing us to identify key transcription factors potentially regulating the gene set that exhibits significant changes with CSS. To assess differential TF activity across samples, we employed the Dorothea (34, 35) and VIPER (36) packages. Specifically, we filtered the Dorothea database to include only high-confidence TF–target interactions (confidence levels A, B, and C) to construct a robust, regulatory network. Virtual Inference of Protein-activity by Enriched Regulon analysis (VIPER) was then applied to our RNA-seq data with parameters such as minsize = 4 and eset.filter = FALSE to calculate normalized TF activity scores based on the expression patterns of each TF’s target genes. These scores, *i.e.*, TF activated (positive values) or repressed (negative values), were used to compare TF activity changes across different disease conditions.

### Data Visualization

We employed the ggplot2 package in R to generate most visualizations, including bar plots, pie plots, volcano plots, box plots, and Sankey diagrams. Workflow diagrams were created with BioRender, network plots with Cytoscape, and pathway diagrams with the pathview package in R. Circos plots and SNP density plots were generated using the circlize and CMplot packages in R, respectively. All code used to generate the figures presented in the manuscript can be found at the following link: https://github.com/digitalproteomes/MMA-MultiOmics-Analysis.

## Results

### Identification of cis-pQTLs and Their Functional Enrichment

**Fig. 1:**
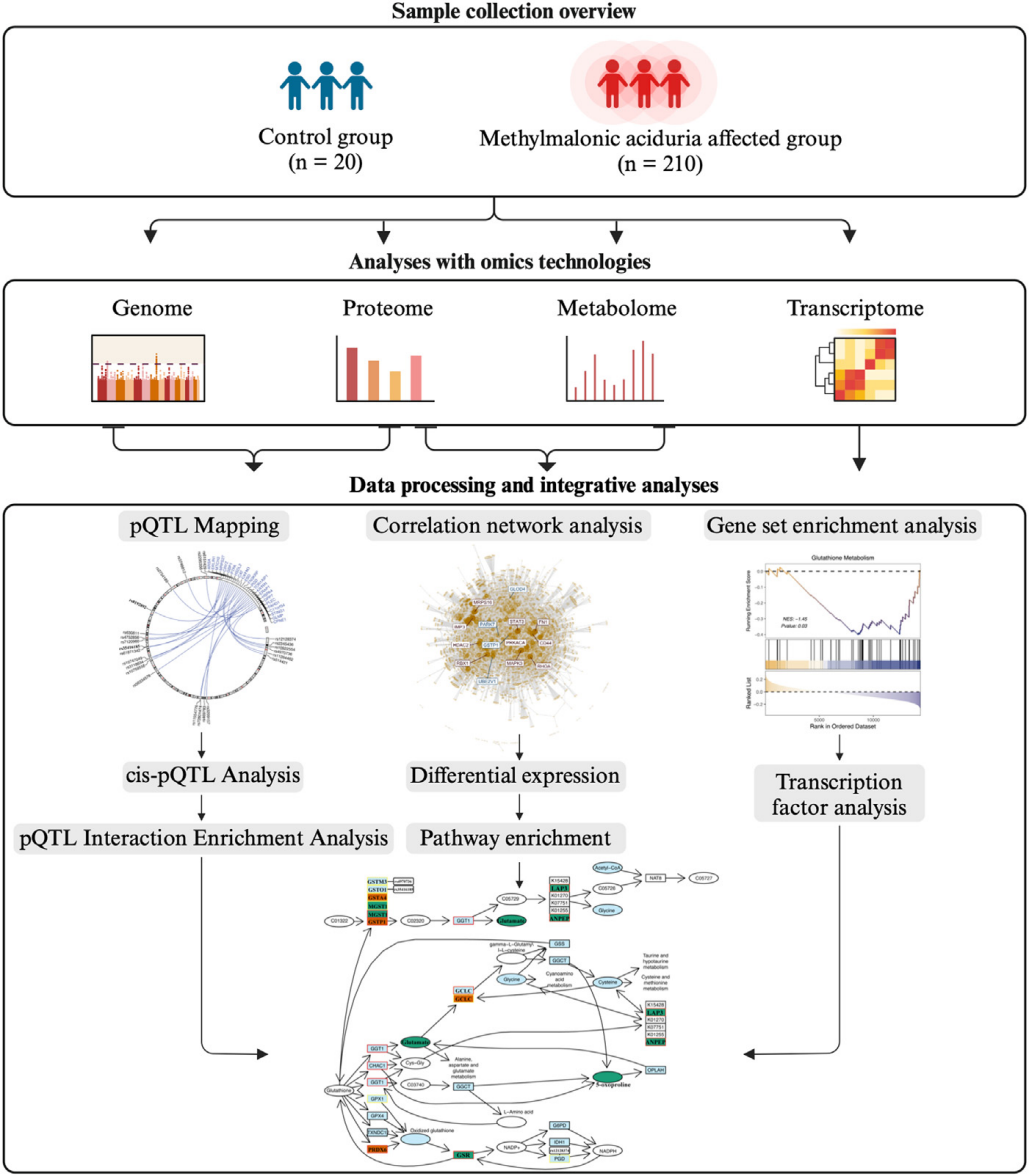
**Workflow-Chart**. The flowchart depicts our analytical workflow for identifying potential contributors and pathways involved in the pathophysiology of MMA. Multi-omics data were previously generated at the genome, transcriptome, proteome, and metabolome levels. These data modalities were integrated through pQTL mapping and enrichment analysis, in conjunction with correlation network and gene set enrichment and transcription factor analysis.

Here, we used a conceptual framework where we first identified potential disease-related pathways using pQTL analysis. Then we integrated evidence for the pathway relevance based on analyses from individual omics’ layers using correlation network analysis as well as correlation with disease severity (Fig. 1). This framework was employed to elucidate pathomechanisms of MMA beyond the ones described in the original publication. The dataset utilized for all subsequent analyses consisted of a previously published cohort of 230 fibroblast samples, comprising those of 20 controls and 210 individuals referred for diagnosis of MMA (for a complete sample annotation, see (2) and supplemental Table S1).

**Fig. 2:**
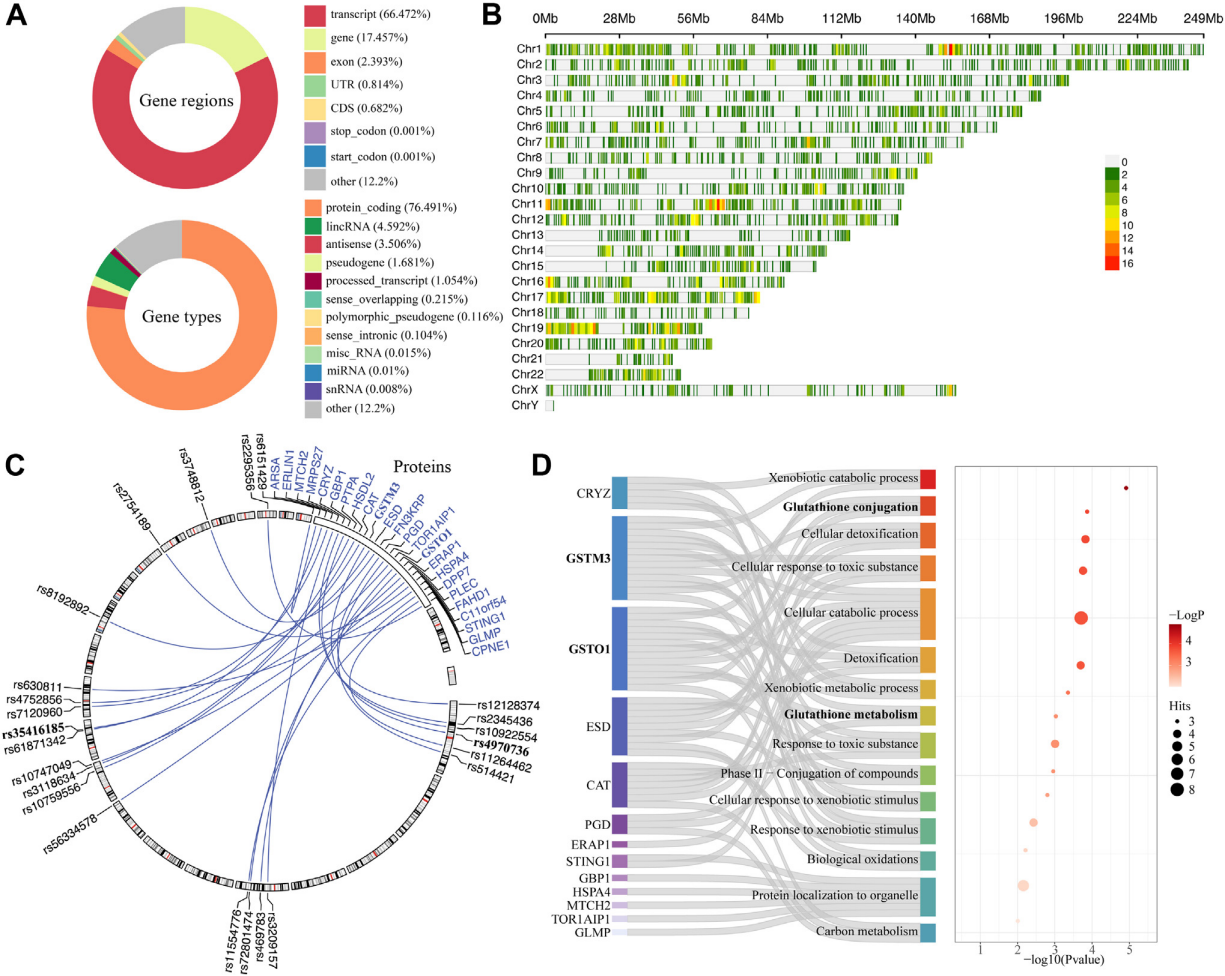
**Cis-pQTL distribution in patients with methylmalonic aciduria**. *A*, distribution of gene regions (*upper panel*) and gene types (*lower panel*) among cis-pQTL variants. *B*, SNP density plot across chromosomes showing the number of SNPs in 1 Mb windows: each horizontal bar corresponds to a human chromosome, the x-axis indicates chromosome length in megabases (Mb), and the color blocks denote the count of SNPs per 1 Mb window, with a gradient from *green* to *red* where darker shades represent a higher SNP density in that genomic region. *C*, Circos plots of cis-pQTL variants: pQTL variants are listed according to their chromosomal location. Proteins with genome-wide significant pQTLs are listed in the right semicircle (*blue color*). *D*, significantly enriched pathways from the pathway enrichment analysis conducted on all proteins in (*C*). The Sankey plot on the *left* displays the enriched pathways and their related proteins. The y-axis depicts enriched pathways. The *dot size* in the *bubble plot* on the *right* indicates the number of protein hits, the color of the dots corresponds to the *p*-value.

We hypothesized that SNPs explaining the variability of nearby proteins across a disease cohort were likely disease-related. Thus, we determined cis-pQTLs for genes and lncRNAs of 176 MMA-related fibroblast samples available in the Forny *et al*. dataset (2) exhibiting no MMUT activity (mut^0^: n = 104), reduced MMUT activity (mut^-^: n = 23), and normal MMUT activity (n = 49) in relation to our proteomic readout. These samples were then compared with those from 14 individuals in the control group (supplemental Fig. S2*A*). First, we used the nominal pass from QTLtools (30, 37) on our data, and 6,951,749 unique SNPs distributed across the entire genome were considered as potential cis-pQTLs for 4373 proteins (supplemental Fig. S2*B*). For each protein, the top cis variant was determined as the cis variant with the strongest association. Then a permutation-based analysis was conducted on the dataset to obtain adjusted *p*-values for the association between protein level and the top cis variants (supplemental Tables S5 and S6). The largest portion of the distribution of gene regions among these cis variants, accounting for 66.472% of the total, is that of the "transcript," followed by the "gene," which constitutes 17.457% (Fig. 2*A*). Other categories, including "Exon," "UTR" (Untranslated Region), "CDS" (Coding Sequence), "Stop Codon" and "Start Codon" represent a smaller proportion. The "Other" category, which includes instances where gene regions could not be mapped, accounts for 12.2% of the total. The distribution of gene biotypes among the cis variants is shown in Figure 2*A*. The distribution of SNPs and proteins along each chromosome is shown in Figure 2*B*.

To identify significant cis-pQTL variants, we applied an adjusted *p*-value threshold of <5e-8 (38, 39), resulting in 24 non-redundant variants each associated with a distinct protein (Fig. 2*C*, supplemental Fig. S3, and supplemental Tables S5 and S6). Among these 24 non-redundant variants, rs4970736 and rs35416185 are associated with the glutathione S-transferase proteins GSTM3 and GSTO1. The glutathione S-transferase (GST) protein family plays a key role in detoxification by catalyzing the conjugation of reduced glutathione with various compounds (40).

Finally, we performed a pathway enrichment analysis of the 24 significant proteins using Metascape (31) to gain insight into their cellular functions. Using Gene Ontology (GO), KEGG pathways, and protein complex databases this analysis identified 15 significantly enriched pathways (Fig. 2*D* and supplemental Table S7). These include processes such as glutathione conjugation, glutathione metabolism, xenobiotic catabolism, cellular detoxification, and response to toxic substances. Notably, glutathione-related pathways, possibly due to glutathione deficiency, have been previously linked to MMA (41). According to our framework, we next wanted to prioritize the list of enriched pathways by gathering evidence from analyses of the individual omics’ layers.

### Identification of Protein Co-expression Modules Altered in MMA Supports cis-pQTL Findings

**Fig. 3:**
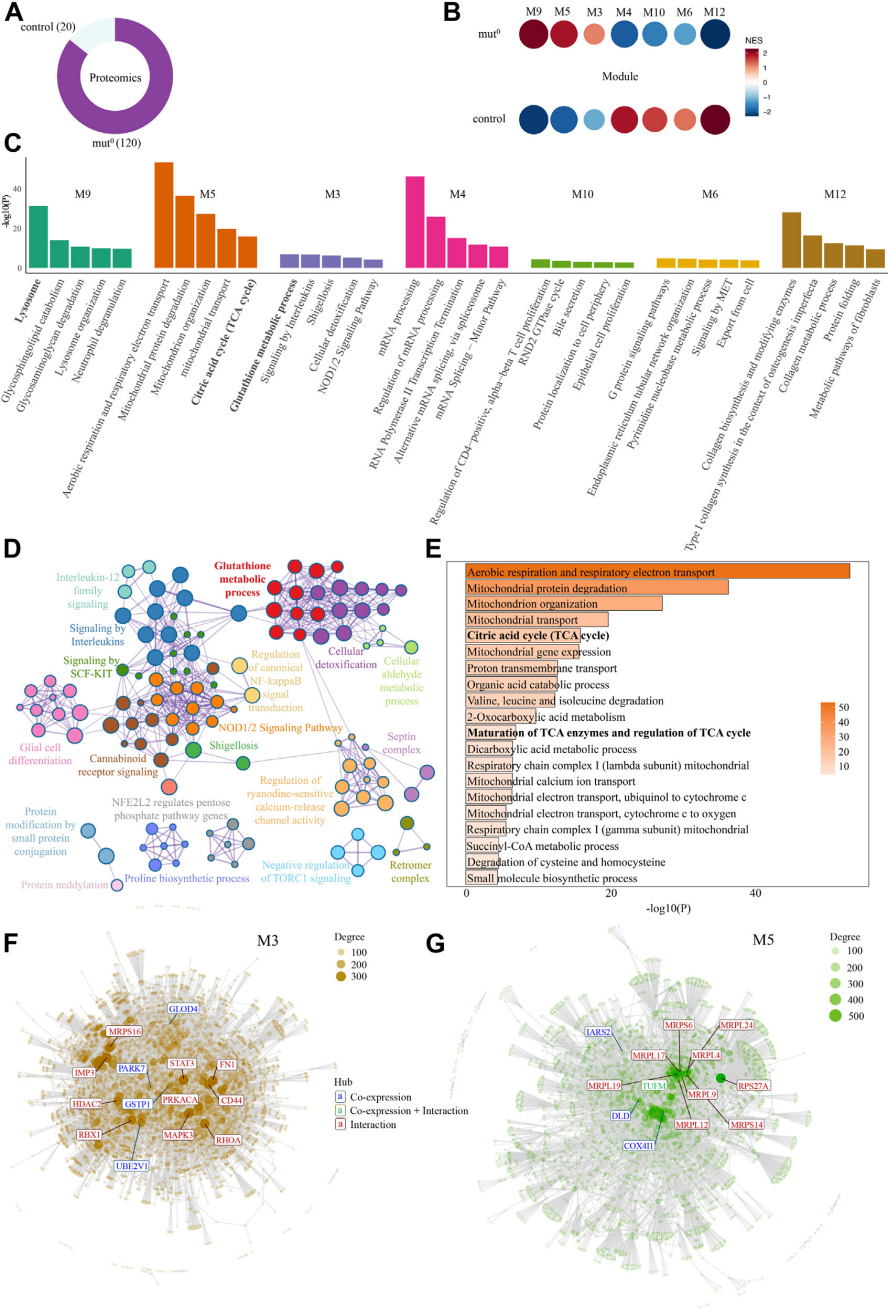
**Protein co-expression modules in patients with methylmalonic aciduria.***A*, schematic showing the number and type of proteomics samples used in our module co-expression analysis. *B*, Bubble heatmap illustrating the results of the module enrichment analysis. *Circle size* and color both represent the normalized enrichment score, as determined by CEMiTool. Only modules with an adjusted *p*-value <0.05 between conditions are shown. *C*, the top five significantly enriched pathways from the pathway enrichment analysis performed on all modules identified in *B* are shown. The height of each bar corresponds to the -log10(p) value. *D*, the enrichment network for proteins in module M3, where each node represents an enriched term. Node sizes correspond to the number of proteins linked to each term, with the top 20 enriched terms highlighted. Different colors distinguish various clusters, and terms within the same cluster are positioned near each other, showing their functional similarity. *E*, Bar chart of the top 20 enriched pathways in the module M5 colored by *p*-value. *F* and *G*, visualization of protein interactions in the co-expression modules M3 and M5, *red* if derived from the interaction file (STRING database), *blue* if proteins are module hubs, and *green* if both conditions apply. Node size reflects the degree of connection strength as determined by CEMiTool. Pathways discussed in the manuscript are indicated in *bold*.

To add further support to our cis-pQTL findings, we next investigated changes in the abundance of protein modules by looking into the protein abundance profiles of 120 individuals with no MMUT activity (mut^0^) and the 20 individuals in the control group (Fig. 3*A*). The CEMiTool (16) computed normalized enrichment scores (NES) identified seven co-expressed modules significantly different between the two classes. Specifically, modules M9, M5, and M3 were enriched in mut^0^, while modules M4, M10, M6, and M12 were enriched in controls (Fig. 3*B*). Pathway enrichment using Metascape (31) associated M9 to lysosomal function, M5 to TCA cycle regulation, and M3 to glutathione metabolism (Fig. 3, *C*–*E* and supplemental Table S8). These findings align with previous research on lysosomal-autophagy dysfunction and TCA disruptions in MMA (2, 42) and support our findings from the cis-pQTL analysis above. The complete list of enriched terms within the seven modules, along with their corresponding -log10(*p*-values), is provided in supplemental Table S9.

To better understand the molecular functions of proteins within the modules, we focused on the central hub proteins as they play key roles in network structure and are obvious targets in disease (43). In module M3, overexpressed in the mut^0^ group, hub proteins include Glutathione S-transferase P (GSTP1), Parkinson disease protein 7 (PARK7), Ubiquitin-conjugating enzyme E2 variant 1 (UBE2V1), and Glyoxalase domain-containing protein 4 (GLOD4) which are linked to glutathione metabolism (Fig. 3*F*). This complementary analysis thus substantiates the cis-pQTL result that the glutathione pathway is dysregulated in MMA. In module M5, which is also overexpressed in the mut^0^ group, key hub proteins such as mitochondrial elongation factor Tu (TUFM) and mitochondrial isoleucine-tRNA ligase (IARS2) are linked to the TCA cycle. This aligns with previous findings from the same dataset, which highlighted the involvement of dihydrolipoamide dehydrogenase (DLD) in metabolic regulation in MMUT-deficient cells (Fig. 3*G*). The protein interactions in the co-expression modules M9, M4, M6, M10, and M12 are provided as supplemental Fig. S4.

### Metabolite Modules Associated with MMA

**Fig. 4:**
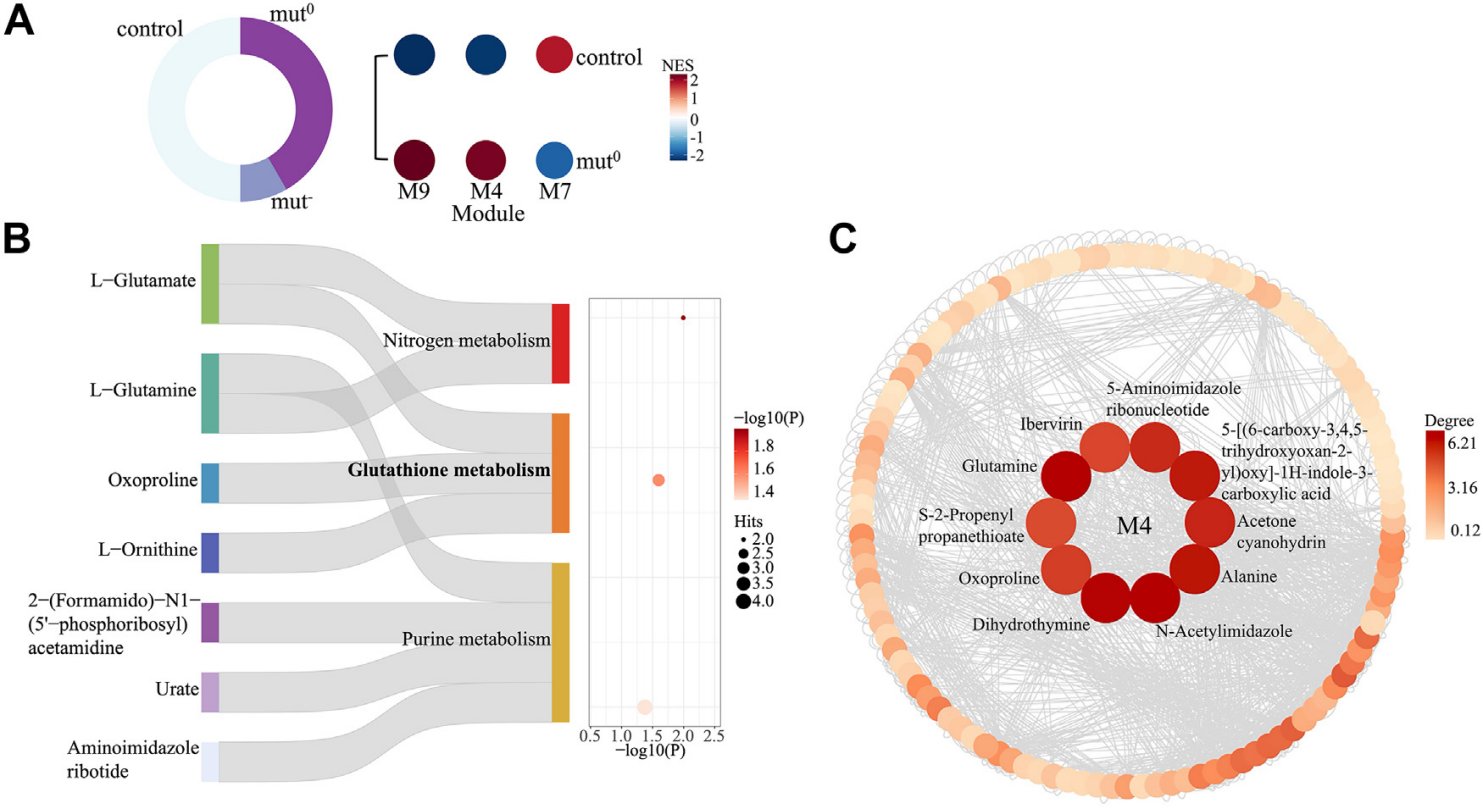
**Metabolites co-expression modules in patients with methylmalonic aciduria**. *A*, *Left side*: schematic drawing showing the number and type of metabolomics samples used in our module co-expression analysis. *Right side*: bubble heatmap depicting the results of module enrichment analysis, highlighting module activities in affected individuals compared to controls. *Circle size* and color both represent the normalized enrichment score, as determined by CEMiTool. Only modules that are significantly different (adjusted *p* < 0.05) across conditions are shown. *B*, significantly enriched pathways from the pathway enrichment analysis conducted on module 4 (M4) identified in (*A*). The Sankey plot on the *left* displays the enriched pathways and their related metabolites. The *dot size* of the bubble plot on the *right* indicates the number of metabolite hits, the color of the dots corresponds to the *p*-value. *C*, all metabolites from M4 are displayed using Cytoscape. The top 10 hub metabolites in M4 are depicted as *red circles* (*inner rim*). The degree of connectivity of all metabolites, as calculated by CEMiTool, is indicated through a color scale ranging from *red* (*high*) to *yellow* (*low*).

Next, we again applied the module expression analysis method by Russo *et al*. (16), this time to metabolomics data. This enabled us to explore differential pathway-level metabolic changes in MMA vs. the control group of the published Forny *et al*. dataset (2) (Fig. 4*A* left). We identified three annotated co-expressed modules: modules M9 and M4 exhibited higher co-expression in the disease group compared to the controls; conversely M7 showed lower co-expression in the disease group (Fig. 4*A* right and supplemental Table S10). Pathway enrichment analysis using MetaboAnalyst (32) for the three modules is shown in Figure 4*B*, supplemental Fig. S5, and supplemental Table S11, with nodes of different colors representing their corresponding enrichment terms. It is noteworthy that module M4 contains metabolites associated with nitrogen metabolism, purine metabolism, and glutathione metabolism. This substantiates the importance of these pathways and corroborates the findings related to glutathione metabolism at the protein and pQTL levels. Glutamate, oxoproline, and ornithine are key metabolites in the glutathione metabolism pathway (as shown in Fig. 4*B*), with oxoproline identified as hub metabolite (Fig. 4*C*). Module M7 contains metabolites associated with lipoic acid and tyrosine metabolism, as shown in supplemental Fig. S5. This is in line with prior findings indicating that in MMUT-deficient cells the alteration of oxoadipate represents one of the most significant metabolic deviations, suggesting possible specific regulatory anomalies in these pathways (2).

### Transcriptomics Gene Sets Associated With MMA

**Fig. 5:**
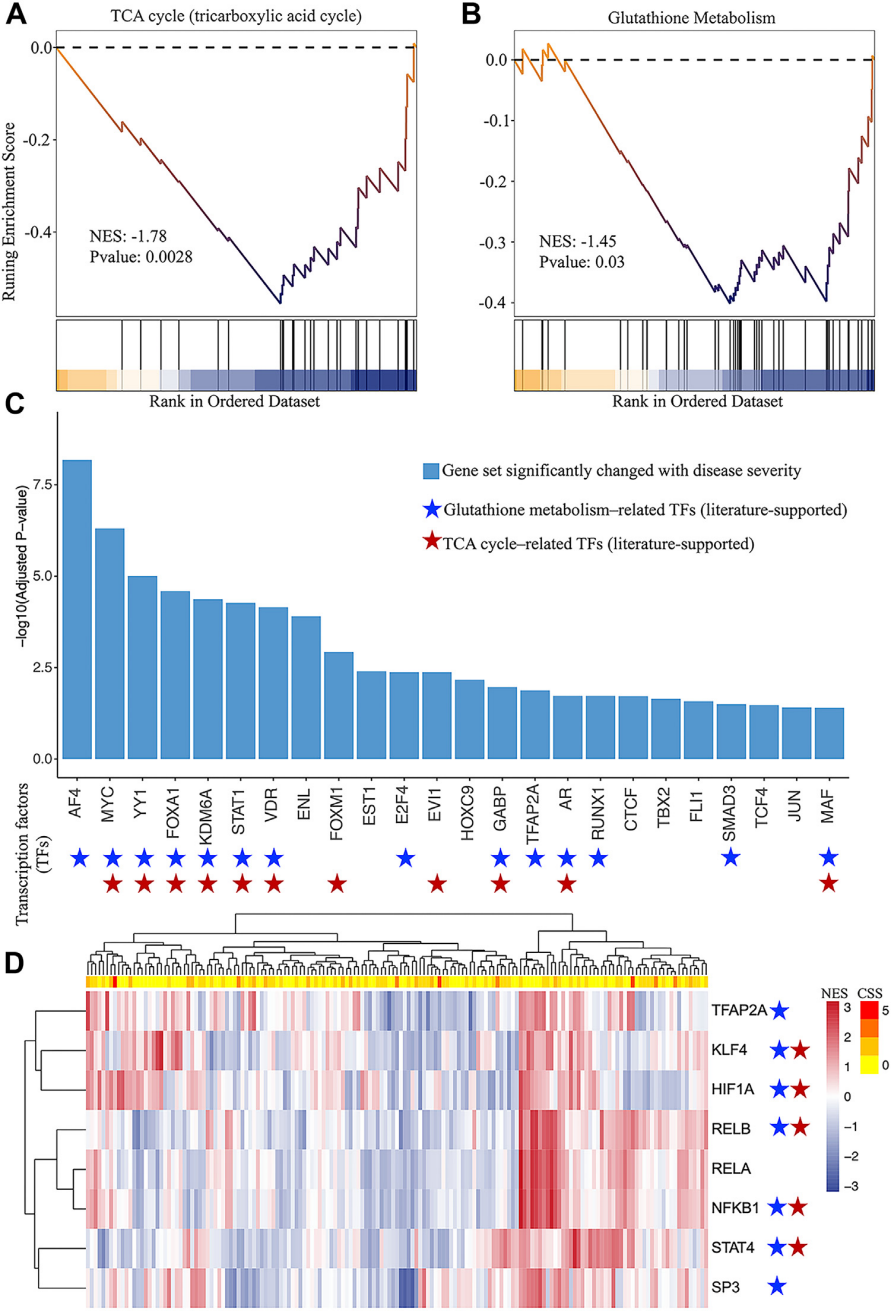
**Gene set enrichment and transcription factor analyses of the transcriptomics dataset in MMA**. Gene Set Enrichment Analysis (GSEA) revealed enrichment in gene sets related to the (*A*) TCA cycle and (*B*) glutathione metabolism pathway based on transcriptomics data. The *top* panel displays the running enrichment score (ES), which reflects the cumulative enrichment of the pathway by ranking the genes in the order of their relevance or impact. The peak ES represents the highest degree of enrichment. The *bottom panel* indicates the gene positions within the ranked gene list. The nominal *p*-value was calculated using the permutation test in GSEA. In *panel* (*C*), the *blue bars* represent the transcription factors (TFs) that showed significant changes in relation to the clinical severity score (CSS). *Blue stars* mark TFs associated with glutathione metabolism, as supported by literature, while *red stars* denote TFs related to the tricarboxylic acid (TCA) cycle, also supported by literature. *D*, the heatmap displays the transcription factor activity scores computed using VIPER, with each row representing a transcription factor and each column representing a sample. *Red* indicates upregulation of TF’s target genes, while *blue* indicates overall downregulation. The CSS for each patient sample is depicted as a gradient from *yellow* to *red*, with deeper colors signifying more severe disease.

We applied Kendall correlation to the available transcriptomics data to associate genes with a clinical severity score (CSS) (2) and conducted a Gene Set Enrichment Analysis (GSEA) to identify significantly enriched, disease-related pathways. GSEA results showed enrichment in gene sets related to the TCA cycle (*p* = 0.0028) (Fig. 5*A*), and this finding aligns with previous results (2). The analysis also showed that the glutathione metabolism pathway was enriched at the transcriptome level (*p* = 0.03) (Fig. 5*B*). Thus, we observed several significant changes in the expression levels of genes involved in glutathione metabolism (supplemental Fig. S6).

**Fig. 6:**
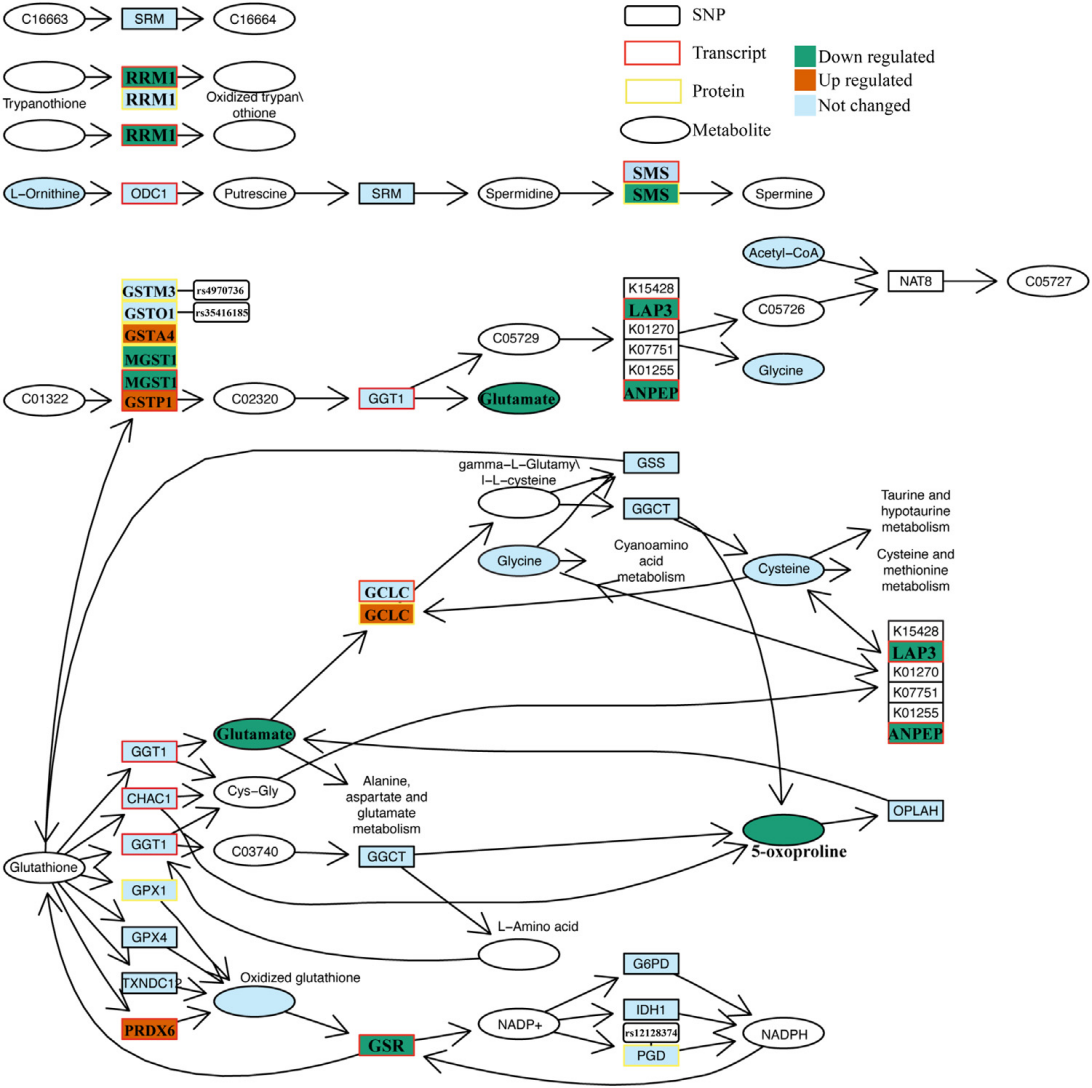
**Glutathione pathway analysis.** An integrated view of the changes in the KEGG glutathione metabolism pathway based on the pQTLs, transcriptomic, proteomic, and metabolomic changes. Ellipses represent metabolites, while squares represent SNPs/transcripts/proteins. Colors indicate the changes between MMA (mut^0^) and the control group (see also supplemental Figs. S3 and S6–S8), with *green* representing downregulation, *orange* representing upregulation, and *blue* representing no significant change.

To further elucidate the regulatory mechanisms driving these pathway disruptions, we performed transcription factor (TF) enrichment and differential activity analyses. Using the ChEA3 database (27), we conducted a TF enrichment analysis on genes that are significantly correlated with the CSS, yielding 24 transcription factors that showed significant enrichment (Fig. 5*C* and supplemental Table S12). Several of these transcription factors are known to play roles in metabolic regulation. For example, the transcription factors AF4 (AF4/FMR2 Family Member 1) (44), MYC (MYC Proto-Oncogene, bHLH Transcription Factor) (45), YY1 (Yin Yang 1) (46), FOXA1 (Forkhead Box A1) (47), KDM6A (Lysine Demethylase 6A) (48), STAT1 (Signal Transducer and Activator of Transcription 1) (49), VDR (Vitamin D Receptor) (50), E2F4 (E2F Transcription Factor 4) (51), GABP (GA Binding Protein) (52), transcription factor AP-2 alpha (TFAP2A) (53), AR (Androgen Receptor) (54), RUNX1 (Runt Related Transcription Factor 1) (55), SMAD3 (SMAD Family Member 3) (56), and MAF (Musculoaponeurotic Fibrosarcoma Oncogene Homolog) (57) have all been associated with glutathione metabolism. Additionally, transcription factors including MYC (58), YY1 (59), FOXA1 (60), KDM6A (61), STAT1 (62), VDR (63), FOXM1 (Forkhead Box M1) (64), EVI1 (Ecotropic Viral Integration Site 1) (65), GABP (66), AR (67), and MAF (68) have been linked to the regulation of the TCA cycle. Our analysis of transcription factor activity using VIPER revealed that several transcription factors, including TFAP2A, Kruppel-like factor 4 (KLF4), hypoxia-inducible factor 1-alpha (HIF1A), RELB proto-oncogene (RELB, an NF-κB subunit), RELA proto-oncogene (RELA, another NF-κB subunit), nuclear factor kappa B subunit 1 (NFKB1), signal transducer and activator of transcription 4 (STAT4), and transcription factor Sp3 (SP3), exhibited significant differences in activity across samples with varying CSS (refer to Fig. 5*D* and supplemental Table S13). Notably, TFAP2A was also identified in our TF enrichment analysis as being associated with glutathione metabolism. Additionally, the transcription factor SP3 has been linked to the regulation of microsomal glutathione transferase (MGST) (69). Previous studies have demonstrated that NF-κB regulates both the core enzymes of the TCA cycle (70) as well as glutathione metabolism. (71, 72). Our data indicates that, within glutathione metabolism, the NF-κB target *GSTP1* (73) is most affected by the differential activity of the transcription factor (supplemental Table S13), which may lead to the significant differences observed in GSTP1 protein expression (Fig. 6).

### Mapping SNP, Transcript, protein, and Metabolite Changes to the Glutathione Pathway

Our analyses on the SNP, transcript, protein, and metabolite levels helped us to prioritize the pathways found in the initial pQTL analysis: all omics’ layers consistently indicated the glutathione metabolism pathway as disease related. To gain an integrated view of these findings we projected our results from the different omics’ layers to the glutathione metabolism KEGG pathway (Fig. 6). Several proteins central to the glutathione metabolism pathway, namely microsomal glutathione S-transferase 1 (MGST1), glutathione S-transferase P (GSTP1), and glutathione S-transferase A4 (GSTA4) from the GST enzyme family, exhibited significant changes in expression when comparing MMA-affected samples to control groups. The previously identified hub protein GSTP1 showed a significant increase in the mut^0^ group compared to the controls (two-sided Wilcoxon rank test, *p* = 0.05, supplemental Fig. S7). In contrast, MGST1 exhibited a significant decrease (*p* = 0.0051) in the mut^0^ group. Additionally, peroxiredoxin-6 (PRDX6), a bifunctional enzyme with glutathione peroxidase activity (74), was also found to be significantly elevated in mut^0^ compared to controls (*p* = 0.028). Also, several significant pQTL-protein pairs related to the glutathione metabolism pathway were identified, namely rs4970736 associated with glutathione S-transferase mu 3(GSTM3), rs35416185 associated with glutathione S-transferase omega 1 (GSTO1), and rs12128374 associated with phosphogluconate dehydrogenase (NADP(+)-dependent, decarboxylating) (PGD). In the metabolomics data, the previously identified hub metabolite oxoproline showed a significant decrease in the MMA samples (*p* = 0.041, supplemental Fig. S8). Furthermore, as previously demonstrated in our research and corroborated in MMUT-KO 293T cells, the glutamate pool size was found to be diminished in MMA fibroblasts (2). In the transcriptomics data, glutathione S-transferase alpha 4 (*GSTA4*, *p* = 0.03) and glutamate-cysteine ligase catalytic subunit (*GCLC p* = 0.0026) showed an increase in the mut^0^ group compared to controls. Consistent with the proteomics data, *MGST1* also exhibited a significant decrease (*p* = 0.00046) in the mut^0^ group. Additionally, glutathione reductase (*GSR*, *p* = 0.043), ribonucleoside-diphosphate reductase large subunit (*RRM1*, *p* = 0.013), cytosol aminopeptidase (*LAP3*, *p* = 0.029), and aminopeptidase N (*ANPEP*, *p* = 0.018) all exhibited a significant decrease in the mut^0^ group. Our integrative analysis approach, anchored in pQTL analysis, collectively, underscores a potential dysregulation of the glutathione pathway in MMA.

## Discussion

Methylmalonic aciduria is an inborn error of metabolism with a complex pathophysiology. The disease involves not just the accumulation of toxic metabolites, but also mitochondrial dysfunction, oxidative stress, and other cellular abnormalities (2, 75, 76). In this study, we used pQTL analysis on an MMA cohort to identify a set of potentially disease-related pathways. Then we ran a series of single omics analyses that explicitly contrast disease groups or severity with omics readouts, to identify disease-related pathways. These analyses include correlative network analysis and GSEA summarized correlation with a disease severity score. Jointly they provided layers of evidence to prioritize the pQTL-identified pathways and position the glutathione pathway as a contributor to the pathomechanisms of MMA. Notably, the relevance of this pathway was missed in the original analysis of the dataset using an integrated analysis based on multi-omics factor analysis and differential expression analysis-based GSEA (2). This exemplifies the importance of FAIR (Findable, Accessible, Interoperable, and Reusable) data principles in scientific research, demonstrating how alternative analytical strategies applied to shared datasets can uncover new biological insights and advance our understanding of complex disease mechanisms.

Glutathione, while synthesized exclusively in the cytosol, is distributed across various cellular compartments, including mitochondria. The mitochondrial glutathione pool maintains a reduction state like that of the cytosol, yet remains kinetically isolated due to the limited transport across the inner mitochondrial membrane. Within the mitochondrial compartment, the glutathione and thioredoxin systems exhibit extensive crosstalk, with both playing crucial roles in eliminating reactive oxygen species (ROS), particularly hydrogen peroxide produced in mitochondria. In individuals affected by MMA, the elevated level of methylmalonic acid in mitochondria impedes the transport of glutathione *via* the mitochondrial dicarboxylate carrier SLC25A10 (77, 78), leading to a redox imbalance and the depletion of mitochondrial antioxidant defenses (79, 80). This inhibition, in conjunction with the accumulation of methylmalonic acid, gives rise to an increase in the production of ROS and reactive nitrogen species (81, 82), thereby promoting oxidative stress (83). Indeed, glutathione metabolism has been shown to be disturbed in the related disorder cobalamin deficiency type C (cblC) (84), in which the production of the cofactor of MMUT is disturbed. Given the critical role of glutathione in protecting against oxidative damage, the targeting of the glutathione pathway could provide an additional potential therapeutic strategy in MMA (82, 85). For example, N-acetylcysteine supplementation may increase glutathione synthesis and benefit affected individuals by reducing oxidative stress and restoring redox balance (86). In addition to treatment, alterations in glutathione metabolism may serve as biomarkers for disease progression, patient stratification, or treatment efficacy for MMA (87).

Next to glutathione metabolism and the TCA cycle, our proteomics data revealed that lysosomal function is also altered in the context of MMA. In a recent study, Costanzo and colleagues demonstrated that lysosomal autophagy dysfunction is a consequence of MMA (42). Lysosomes play a key role in maintaining metabolic balance through the degradation and recycling of macromolecules. However, the accumulation of metabolites such as methylmalonic acid can result in an overload of the system, which may impair its function. By studying fibroblasts from two mut^0^ patients, Costanzo and colleagues observed a marked reduction in the expression of the LAMP2 protein and its transcript, which mediates the fusion of lysosomes with autophagosomes. Interestingly, other studies have also linked LAMP2 efficiency to oxidative stress, including ROS, through the reduction of cytosolic cysteine concentration, resulting in low glutathione (88). This suggests a potential mechanistic interaction between the two pathways.

In this study, we used MMA to showcase how the integration of multi-omics data provides a robust framework for prioritizing molecular pathways implicated in disorders with complex pathophysiologies. Indeed, the apparent role of the glutathione pathway in MMA pathogenesis was supported by evidence from multiple omics levels. At the gene level, the pQTL analysis revealed specific variants associated with altered expression levels in proteins involved in glutathione metabolism. This finding was supported by correlation network analysis of both proteomic and metabolomic data, which demonstrated significant changes between diseased and controls in the abundance of proteins and metabolite modules related to glutathione metabolism. A gene set enrichment analysis correlating the transcript levels with disease severity further substantiated the dysregulation of the glutathione metabolism in MMA. Furthermore, our transcription factor activity analysis revealed that NF-κB may serve as a crucial driver of glutathione metabolism dysregulation, a finding supported by significant changes in its target GSTP1. To substantiate this finding, further experimental validation such as ChIP-Seq will be required. By leveraging the complementary nature of the different omics technologies, we were able to strengthen the evidence linking glutathione metabolism dysregulation to MMA. This multi-level validation underscores the power of integrative approaches in unraveling complex disease mechanisms and will increasingly play an important role as more multi-omic clinical datasets become available.

In this study, we propose that SNPs affecting variability in nearby protein levels—analyzed in a cohort reflecting disease heterogeneity—are likely linked to disease pathology. This hypothesis aligns with prior evidence demonstrating associations between SNPs, protein expression, and disease outcomes (89, 90, 91, 92). While our cohort’s limited size increases the risk of confounding effects (*e.g.*, natural genetic variation unrelated to disease status), we mitigated potential false positives by cross-validating pQTL findings with multi-omics data. Further validation in larger, more diverse cohorts is needed to confirm causality and generalize these associations between SNPs and disease.

## Conclusion

In this study, we harnessed the power of multi-omics data integration to shed light on the intricate pathomechanisms of MMA. Together our findings suggest that the glutathione pathway is dysregulated in MMA. Our study highlights the importance of a holistic approach to understanding complex pathomechanisms. The integrative analysis approach pursued here offers a framework for unraveling disease mechanisms and has broad applicability to other metabolic diseases, paving the way for future research and therapeutic interventions.

## Data Availability

Due to ethical restrictions, access to the raw genomic and transcriptomic data is restricted however can be made available upon reasonable request to Sean Froese (University Children’s Hospital Zurich, sean.froese@kispi.uzh.ch) within 3 months following an established data transfer, use agreement, and ethical approval. The MS proteomics data have been submitted to the ProteomeXchange Consortium *via* the MassIVE partner repository under dataset identifiers MSV000088791 and PXD038225. The raw metabolomics MS data for human fibroblast measurements are also available in the MassIVE repository under dataset identifier MSV000089082. Source data are included with this publication as supplemental information.

## Code Availability

Code for all analyses done in this project is available on Github: https://github.com/digitalproteomes/MMA-MultiOmics-Analysis.

## Supplemental data

This article contains supplemental data (2).

## Conflict of interest

The authors declare that they have no conflicts of interest with the contents of this article.
