# Supplementary material for: A Multi-Omics Framework for Decoding Disease Mechanisms: Insights From Methylmalonic Aciduria
Source: Mol Cell Proteomics. 2025 May 26;24(7):100998. doi: 10.1016/j.mcpro.2025.100998 (PMC12226375; doi:10.1016/j.mcpro.2025.100998)
Supplement: Supplemental data [file mmc14.docx]

#### Supplemental Data

# A Multi-Omics Framework for Decoding Disease Mechanisms: Insights from Methylmalonic Aciduria

Jianbo Fu^1-3^, Vito R.T. Zanotelli^4^, Cedric Howald^5,6^, Nylsa Chammartin^4-6^, Ilya Kolpakov^5,6^, Ioannis Xenarios^5,6^, D. Sean Froese^4^, Bernd Wollscheid^1,2^, Patrick G.A. Pedrioli^1-3^ and Sandra Goetze^1-3*^

**Supplemental Tables (excel spreadsheets)**

**Supplemental Table S1** Patient sample annotation.

**Supplemental Table S2** Transcriptomics source data.

**Supplemental Table S3** Protein abundance data**.**

**Supplemental Table S4** Metabolomics source data.

**Supplemental Table S5 and S6** Permutation results calculated using QTLtools cis-pQTL analysis and variant annotation.

**Supplemental Table S7** Significant cis-pQTLs associated with protein enrichment analysis.

**Supplemental Table S8** Proteins included in each module identified by CEMiTool.

**Supplemental Table S9** Enrichment analysis for modules M3, M4, M5, M6, M9, M10 and M12 from proteomics data.

**Supplemental Table S10** Metabolites included in each module identified by CEMiTool.

**Supplemental Table S11** Pathway analysis for modules M4, M7, and M9 from metabolomics data.

**Supplemental Table S12** Transcription factor enrichment analysis results for the gene sets that changed significantly with CSS.

**Supplemental Table S13** Analysis of VIPER-inferred transcription factor activity in relation to disease severity (FDR-adjusted) and the target genes associated with transcription factors that show significantly altered activity.

#### Supplemental Figures

####
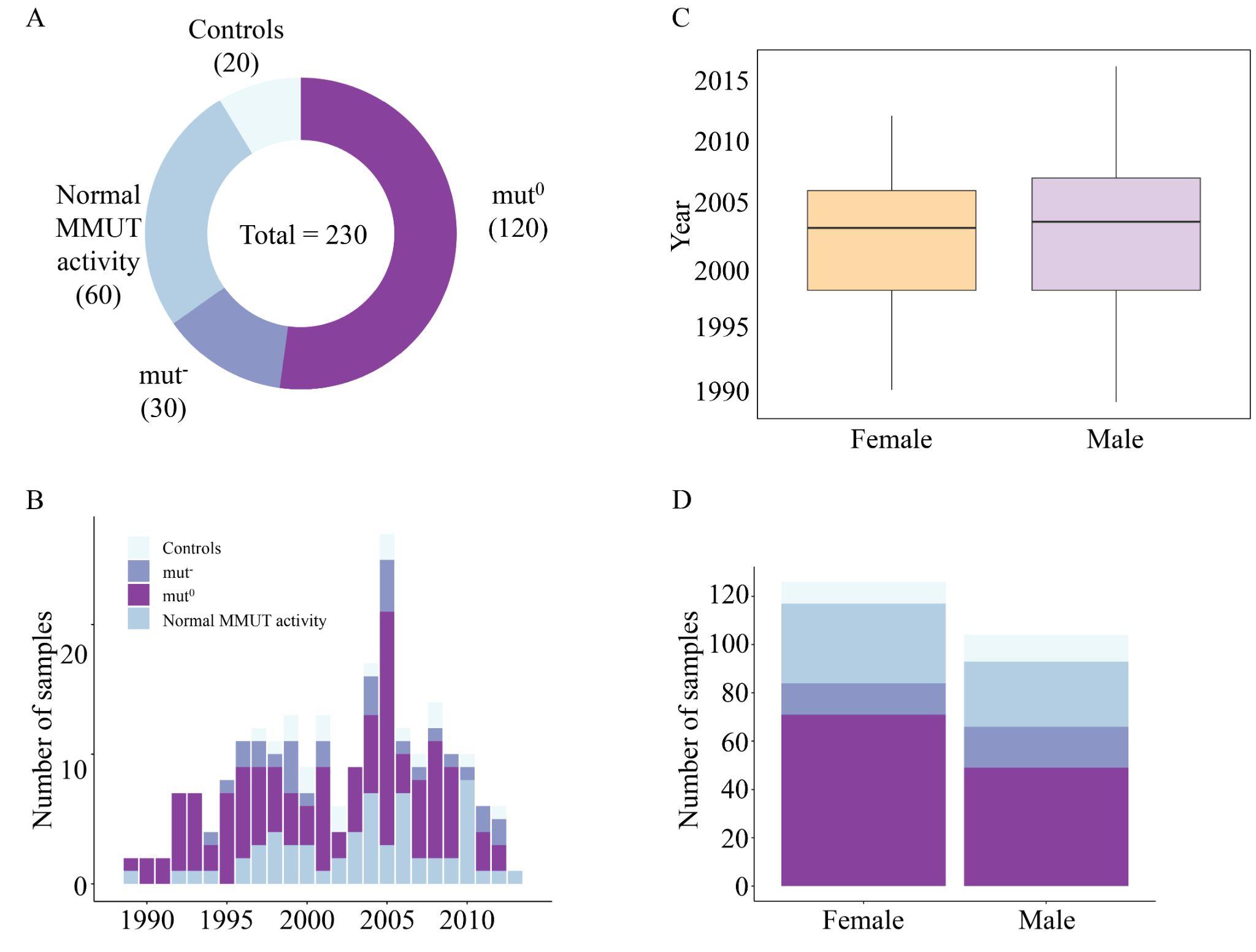


**Supplemental Figure S1 MMA patient cohort overview.** (A) The sample cohort consists of 210 fibroblasts from MMA patients (120 without MMUT activity (mut^0^), 30 with reduced MMUT activity (mut^-^), and 60 suffering from MMA despite normal MMUT activity), and 20 from healthy controls (11 male, 9 female). (B) The fibroblasts from this European patient cohort were collected over a time period of 25 years. (C,D) show the distribution of sex over time and disease phenotype.


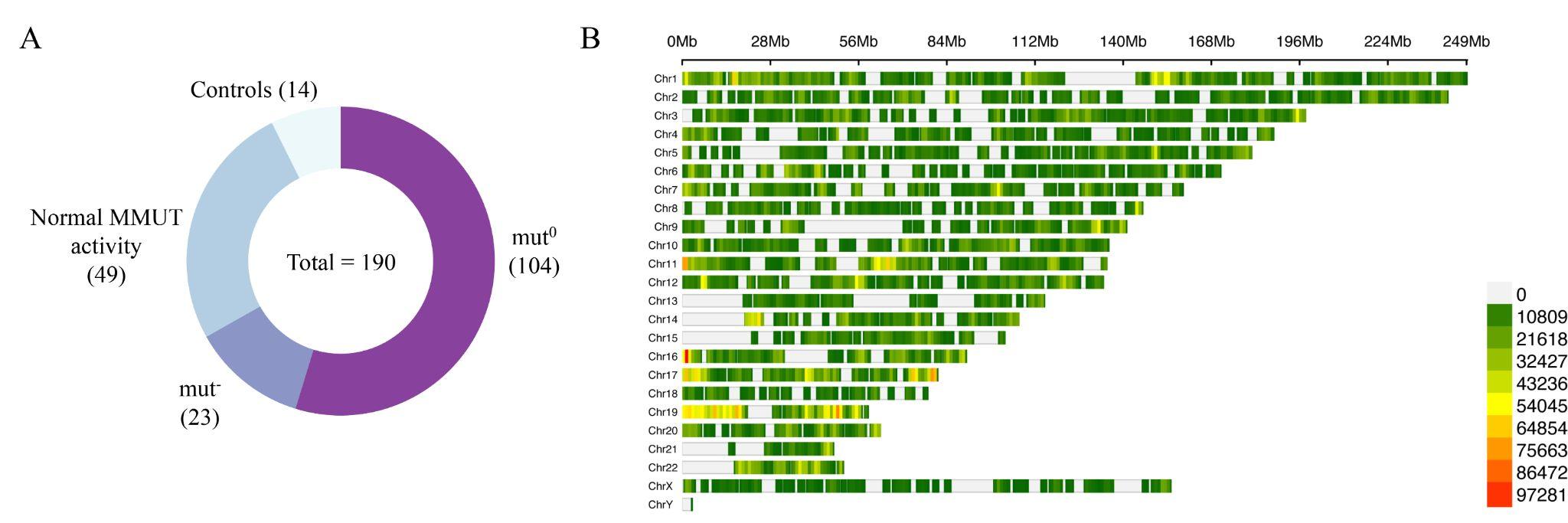


**Supplemental Figure S2 Distribution of unique SNPs across the genome**

(A) Schematic drawing showing the number and type of samples used in our cis-pQTLs analysis. (B)The plot shows the SNP density across chromosomes within a 1 Mb window size. The horizontal axis indicates chromosome length (Mb), with different colors representing SNP density. Data were calculated using the nominal pass from QTLtools.


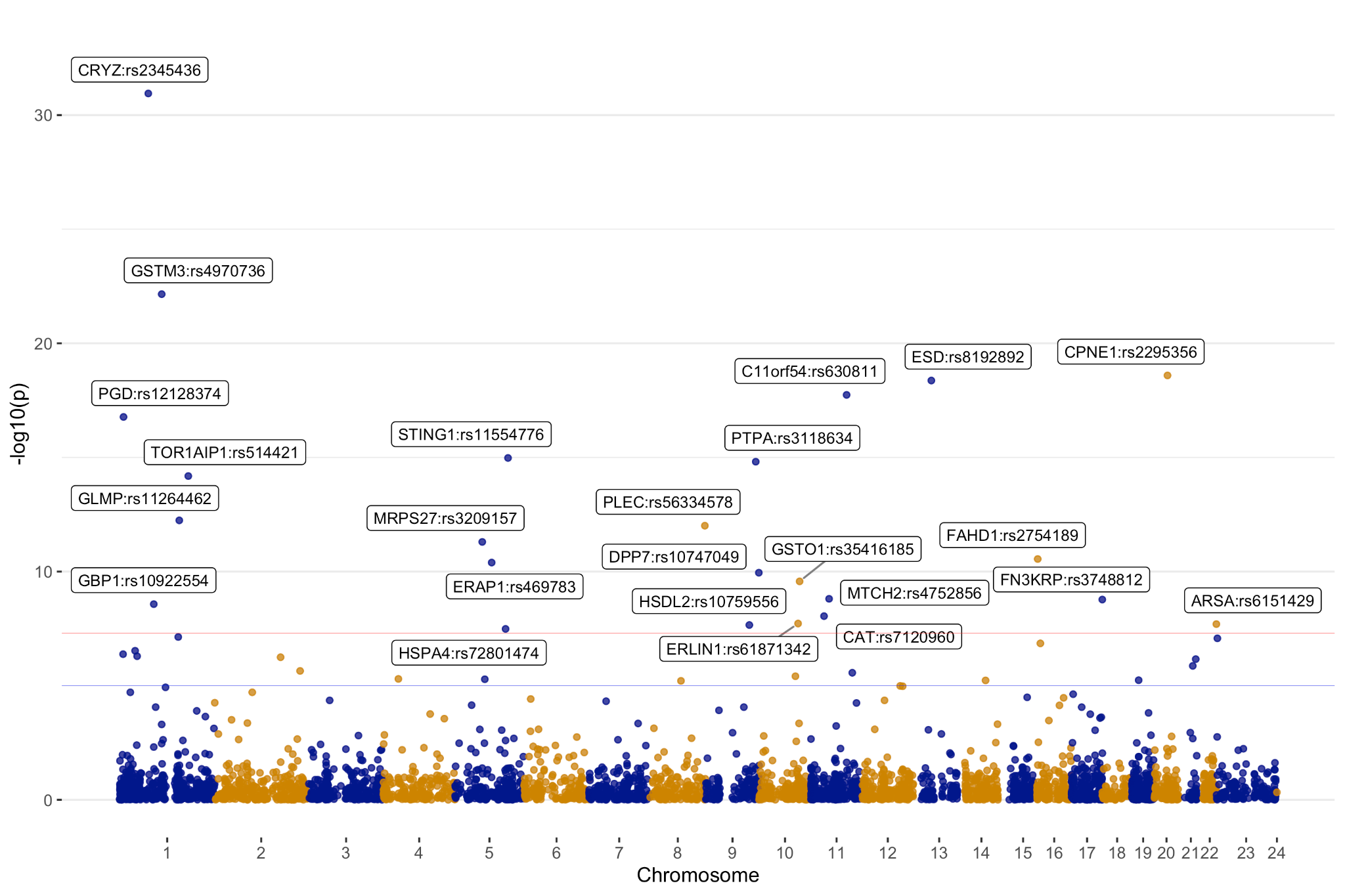


**Supplemental Figure S3 Manhattan plot of cis-pQTL association analysis.**

The x-axis provides chromosomal positions and the y-axis shows -log10 p-values. The red line indicates the genome-wide significance threshold (p=5.0×10^−8^) for cis-pQTLs.

**
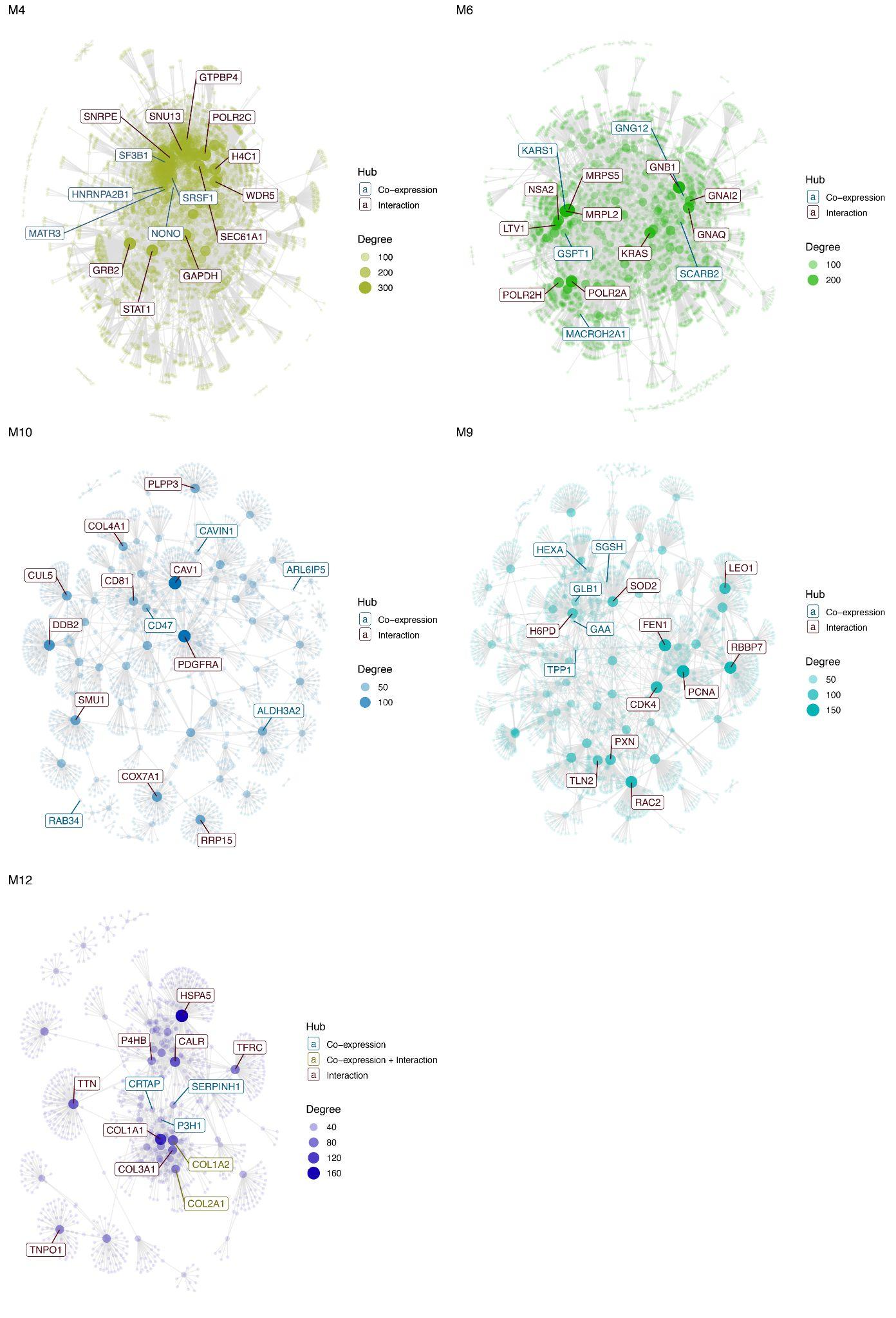
**

**Supplemental Figure S4 Additional protein co-expression modules.**

Visualization of protein interactions in the co-expression modules M4, M6, M9, M10, and M12. Protein names are colored red if interactions were inferred from the interaction file (STRING database), blue if proteins were module hubs, and green if both conditions apply. Node size reflects connection strength degree as determined by CEMiTool. Pathways discussed in the manuscript are shown in bold.


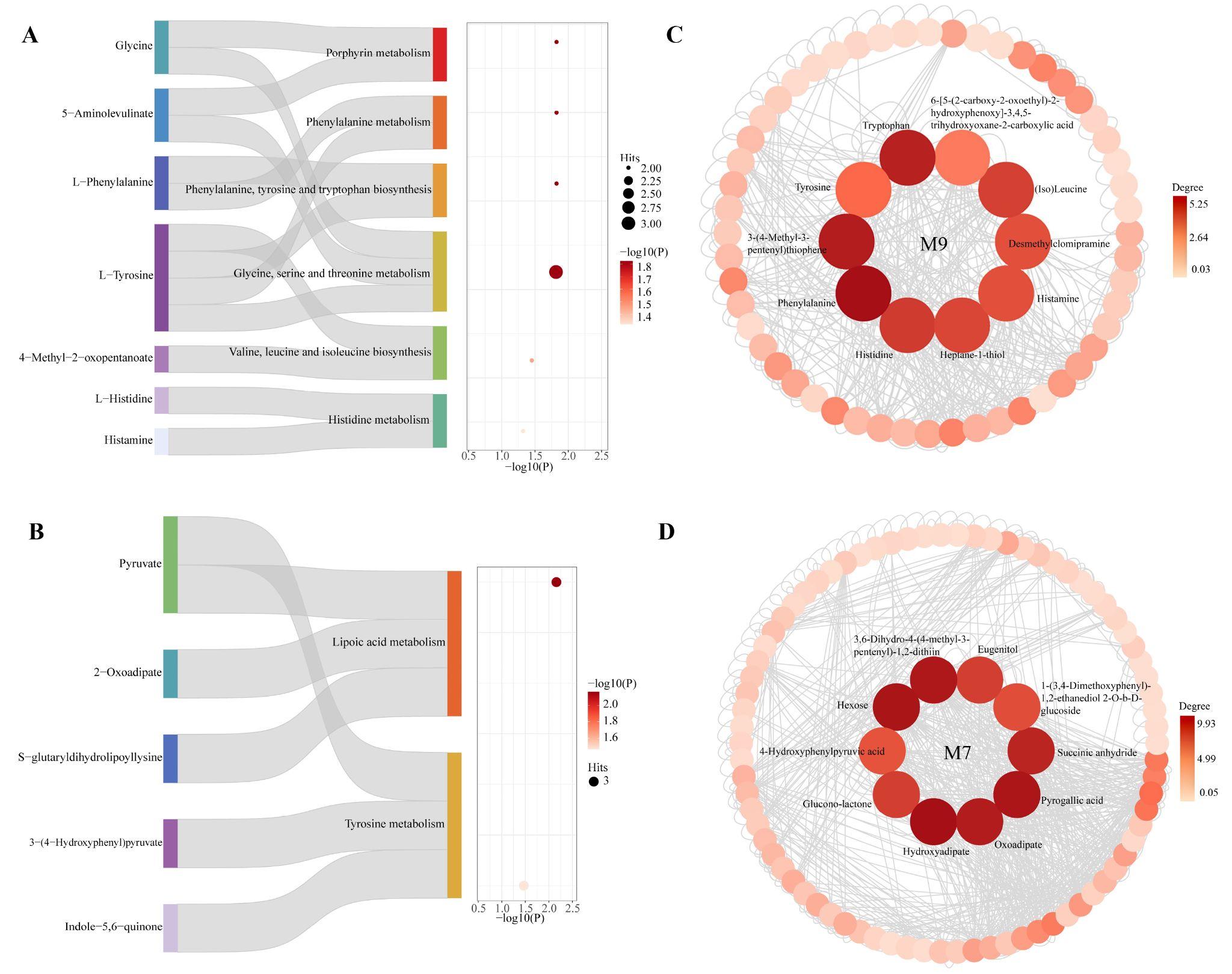


**Supplemental Figure S5 Additional metabolite co-expression modules.**

​​(A, B) depict two significantly enriched pathways from the pathway enrichment analysis conducted on modules M9 and M7 identified for metabolites in Fig. 4A. The y-axis represents enriched pathways. Dot size of the bubble plot on the right indicates the number of metabolite hits, the color of the dots corresponds to the p-value. The Sankey plot on the left displays the enriched pathways and their related metabolites from the corresponding modules. (C, D) All metabolites of M9 and M7 are depicted. Metabolites with the highest number of connections in the network are shown as hub metabolites (top 10). The degree of connectivity of all metabolites, as calculated by CEMiTool, is indicated through a color scale ranging from red (high) to yellow (low).


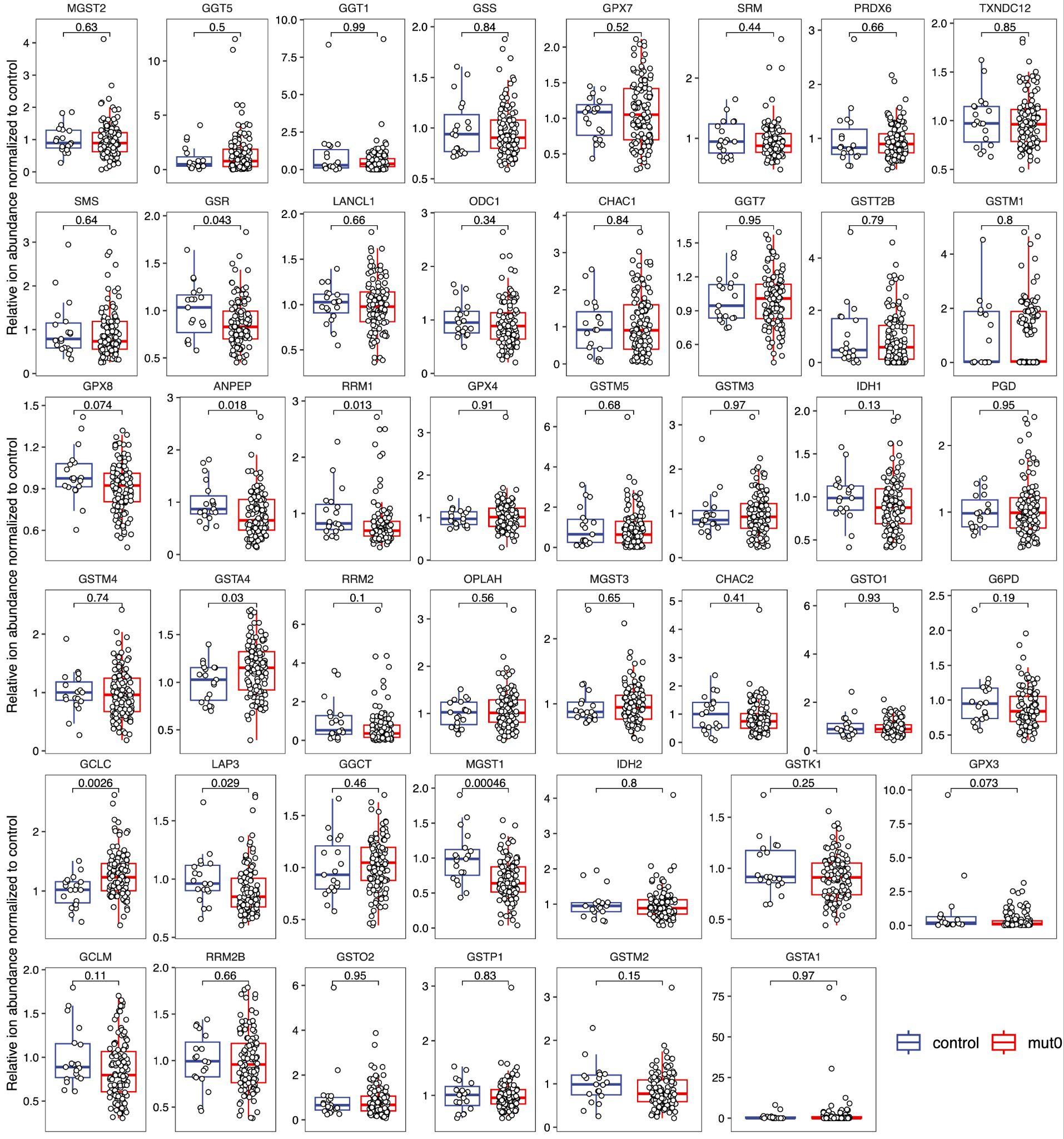


**Supplemental Figure S6 Changes in the levels of transcripts involved in the glutathione metabolism.**

The boxplots illustrate the variations in the abundance of transcripts involved in the glutathione metabolism, compared between control and mut^0^. All p-values are calculated by the Wilcoxon rank test, two-sided.


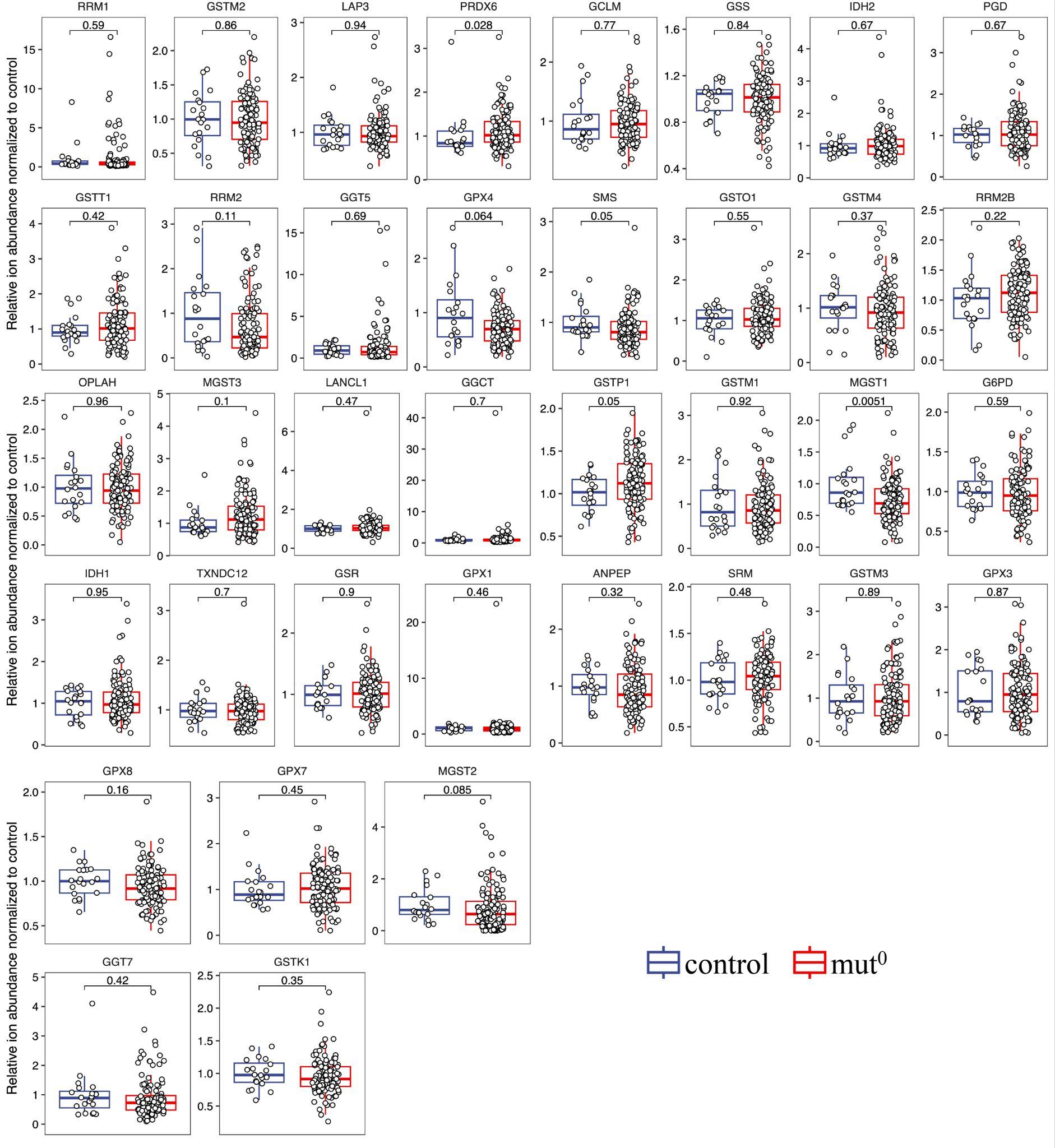


**Supplemental Figure S7 Changes in the levels of proteins involved in glutathione metabolism.**

These boxplots illustrate the abundance variations of proteins involved in glutathione metabolism, compared between control and mut^0^. All p-values are calculated by the Wilcoxon rank test, two-sided.


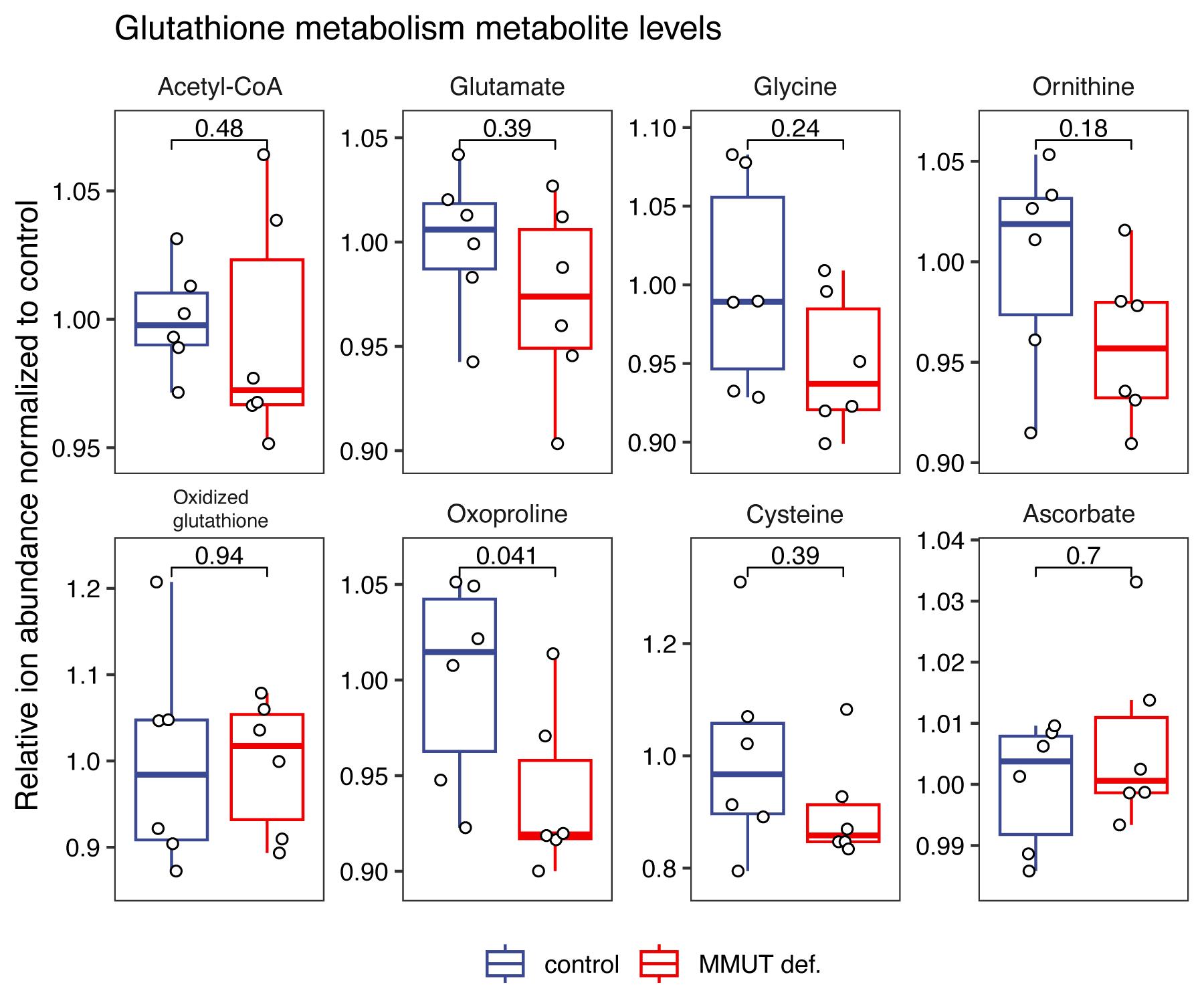


**Supplemental Figure S8. Changes in metabolite levels within the glutathione metabolism.**

The boxplots illustrate the variations in total ion current of metabolites involved in glutathione metabolism, compared between control and mut^0^. Each group consisted of six biological replicates, with technical replicates aggregated to represent a single data point per cell line. All p-values are calculated by the Wilcoxon rank test, two-sided.
